# Invigorating spleen, replenishing qi and tonifying kidney method treatment of traditional Chinese medicine for myasthenia gravis: A protocol for systematic review and meta-analysis

**DOI:** 10.1097/MD.0000000000032285

**Published:** 2022-12-16

**Authors:** Miao Liu, Hongan Wang, Zhiguo Lv, Jing Lu, Dongmei Zhang, Zhiyue Zhu, Chunwei Wu, Ye Tian, Chaoqun Song, Tong Wu, Li Liu, Xinzhi Chen

**Affiliations:** a Changchun University of Chinese Medicine, Jilin Province, China; b Affiliated Hospital of Changchun University of Chinese Medicine, Jilin Province, China; c The First Clinical Hospital Research Institute of Jilin Academy of Chinese Medicine, Jilin Province, China.

**Keywords:** invigorating spleen, replenishing qi and tonifying Kidney, protocol, replenishing qi and tonifying kidney, traditional Chinese medicine

## Abstract

Myasthenia gravis (MG) is an autoimmune disease related to the production of autoantibodies. It is mediated by antibodies against acetylcholine receptor (AChR), muscle specific kinase (MuSK) or other AChR related proteins in the postsynaptic muscle membrane, which interfere with the transmission of signals at the neuromuscular junction, resulting in clinical symptoms of skeletal muscle weakness and fatigue, leading to the occurrence and development of MG. At present, the incidence rate of Mg is increasing year by year. At present, the method of invigorating spleen, replenishing qi and tonifying kidney in traditional Chinese medicine has been widely used in the clinical treatment of MG, and the effect is good. The purpose of this study was to systematically evaluate the efficacy and safety of the method of invigorating the spleen, supplementing qi and tonifying the kidney in the treatment of MG.

**Methods:**

We will search from the following eight databases: PubMed, Cochrane Library, EMBASE, Web of Science, CNKI, Sinomed, Wanfang, and Vip. All randomized controlled trial (RCT) literature has been searched and classified since the establishment of the database to date. In this study, two researchers independently screened and evaluated the quality of the retrieved literature. Cochrane risk bias assessment tool was used to evaluate the risk of bias. The meta-analysis uses RevMan 5.3 software provided by Cochrane Collaboration Network for meta analysis.

**Results:**

This study compared the main outcome indicators: clinical response rate, recurrence rate, incidence of adverse reactions, quantitative myasthenia gravis score (QMG). Secondary outcomes were clinical absolute score, quality of life score (QOL), levels of inflammatory factors such as IL-6, IL-10, and serum acetylcholine receptor antibody (AChR-Ab) levels.

**Conclusion:**

The purpose of this study was to evaluate the efficacy and safety of the method of invigorating the spleen, supplementing qi and tonifying the kidney in treating MG, and to provide evidence based medicine.

## 1. Introduction

Myasthenia gravis (MG) is an autoimmune disease with acquired neuromuscular junction (NMJ) transmission disorder mediated by autoantibodies. The most common subtype feature is the acetylcholine receptor antibody (AchR-Ab) against the nicotinic acetylcholine receptor (AChR) at the neuromuscular junction.^[1]^ The main clinical manifestations were blepharoptosis, limb asthenia, muscle wasting, dysphagia, etc. The symptoms became worse after activities. Western medicine usually adopts glucocorticoid, immunosuppressant, cholinesterase inhibitor, plasma exchange, thymectomy and other methods.^[2]^ Although they have curative effects, they have certain adverse reactions after long-term use.

In recent years, Chinese medicine has played a pivotal role in the prevention and treatment of diseases. MG, an autoimmune nervous system disease, is characterized by recurrent attacks and a prolonged course of illness. According to Chinese medicine, MG belongs to the category of “impotence evidence.” However, according to its clinical manifestations and the different stages of the disease, combined with the traditional Western medicine typology, it can belong to other pathologies in Chinese medicine. In TCM, “lid wasting,” “ptosis,” and “optic disorder” are simple ptosis and diplopia of the eye muscles; in TCM, “head tilt” is for generalized muscle weakness with neck weakness and head lifting; “atmospheric subsidence” is for respite oratory difficulty to respiratory muscle paralysis in all types of muscle weakness.^[3]^ Among the above-mentioned types, patients with MG have mostly spleen and kidney yang deficiency, so TCM practitioners often use the method of invigorating spleen and invigorating qi and tonifying kidney to treat MG as an adjunct to treatment, and often achieve better clinical results. However, there is a lack of evidence-based medical evidence on the effect of Chinese herbal medicines for invigorating spleen and invigorating qi and tonifying kidney on the therapeutic effect of MG. The purpose of this study was to conduct a systematic evaluation of the literature and to analyze the efficacy and safety of spleen-enhancing, qi-supplementing, kidney-supplementing herbal medicines on MG.

## 2. Methods

### 2.1. Agreement registration

The review has been registered in the International Prospective Register of Systematic Reviews (PROSPERO) with the registration number of CRD42022372272. The protocol will be reported in strict accordance with the statement of the Preferred Reporting Project Guidance (PRISMA-P) for the systematic review and meta-analysis programme^[4]^ and the Cochrane Intervention System Evaluation Manual.^[5]^

### 2.2. Qualification criteria

#### 2.2..1. Type of study.

We will include the literature of randomized controlled trials (RCTs) of Chinese herbal medicines for invigorating spleen and invigorating qi and tonifying kidney in treating MG. And we will exclude criteria such as reviews, probability, cohorts, case reports, dissertations, clinical guidelines, and studies without sufficient information on randomized methods or procedures.

#### 2.2..2. Participants.

The patients should meet the internationally recognized diagnostic criteria for MG and be definitely diagnosed as MG, excluding MG patients caused by congenital, drug and other factors, as well as patients with serious primary diseases, autoimmune diseases or mental diseases. Patients are not restricted by race, region, gender, age, background, course of disease and other factors.

#### 2.2..3. Intervention.

We will focus on trials that use the herbs for invigorating spleen and invigorating qi and tonifying kidney at any dose and in any regimen as an intervention. The control group is routinely given Western medicine, including cholinesterase inhibitors, glucocorticoids, and immunosuppressants, alone or in combination, or placebo.

In the intervention group, based on the western medical treatment in the control group, Chinese herbal medicines were applied to invigorating spleen and invigorating qi and tonifying kidney, with no restriction on the specific dosage form and dose. The shortest duration of treatment should be four weeks.

#### 2.2..4. Type of outcomes.

Main outcome measures: quantitative score of myasthenia gravis (QMG); recurrence rate.

Secondary outcome measures: the level of serum AchR-Ab; the levels of inflammatory factors such as IL-6 and IL-10; clinical absolute score; quality of life score (QOL); incidence rate of adverse events.

### 2.3. Search methods for screening relevant research

We will search from the following 8 databases: PubMed, Cochrane Library, EMBASE, Web of Science, CNKI, Sinomed, Wanfang, and Wipu. We will explore the literature of all RCTs of Chinese herbal medicines for invigorating spleen and invigorating qi and tonifying kidney in the treatment of MG since the establishment of the library. In the month when the article is to be completed, the literature will be reviewed again to observe whether there are new relevant literatures. Key search terms (MeSH and Free words) used for our searches are “myasthenia gravis” or “invigorating spleen and invigorating qi and tonifying kidney,” or “Traditional Chinese Medicine” and “RCTs,” etc. In addition, standard search terms for RCTs will be used when possible. There are no planned restrictions in the search strategy to prevent overlooking important studies that are not correctly classified in the respective bibliographic databases. All databases will be searched without time constraints from the very beginning of the database.

### 2.4. Study selection and data extraction

**Figure 1.**
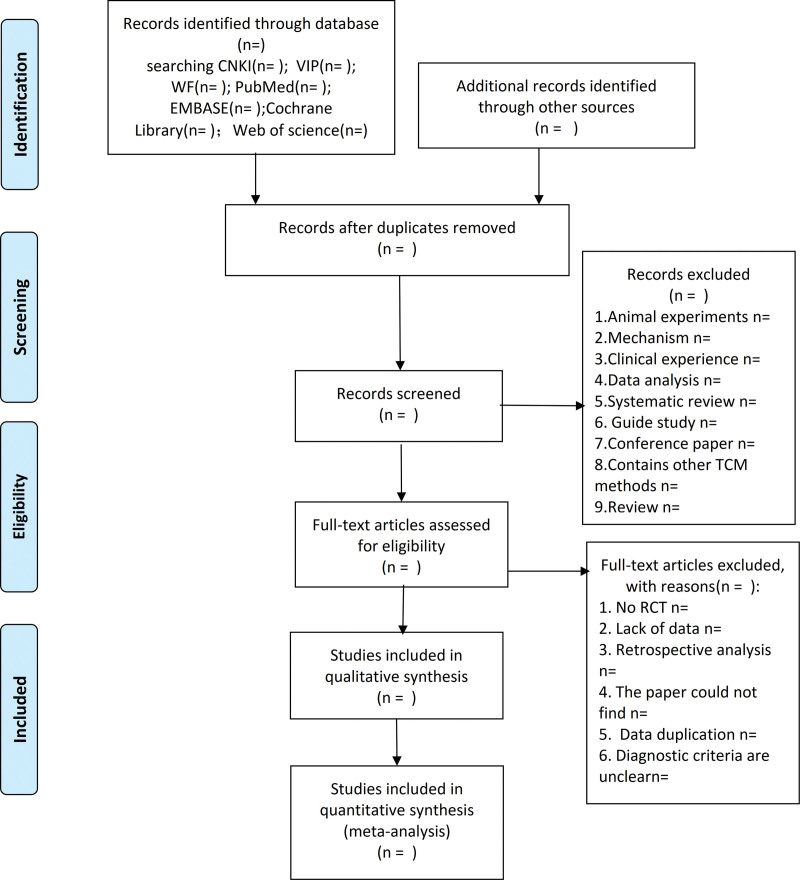
Flow diagram of study selection process.

Two researchers (ZYZ and CQS) will independently review literature screening results and perform data extraction. This task will include entering the following information in the data extraction form: basic information of the study (researcher name, study title, year of publication, country, language, publication status); study characteristics (sample size, source of cases, age, course of disease, diagnosis Criteria, Inclusion Criteria, Exclusion Criteria); Interventions and Controls; Study Methods (Generation of Randomization, Allocation Concealment, Blinding, Baseline Comparability, Loss of Follow-up). The measurement data will be used to determine the meta-analysis outcome index. Disagreements are resolved by discussion or mediated by a third party (YT). The final selection process will follow the Guidelines for Preferred Reporting Items for the Protocol for Systematic Review and Meta-Analysis (PRISMA-P) guidelines, as shown in Figure 1.

### 2.5. Risk of bias

The methodological quality of RCT will be independently assessed by two research members (TY and WBT) using the Cochrane risk of bias (ROB) tool. The following seven criteria will be evaluated: random sequence generation, allocation concealment, blinding of participants and personnel, blinding of result evaluators, incomplete result data, selective reporting, and other deviations. The quality assessment results of each project will be divided into three levels: low risk, unknown risk or high risk. Any differences between the reviewers shall be resolved through discussion or by seeking advice from the Third Party Reviewer (ZDM).

### 2.6. Missing data management

We will contact the original authors of missing data papers by email or phone and wait for a response from the authors after contact. Papers containing incomplete data were excluded from the final analysis if missing data were not available.

### 2.7. Statistical analysis

#### 2.7.1. Meta-analysis.

Data will be statistically analyzed using RevMan Version 5.3. Copenhagen: The Nordic Cochrane Centre, The Cochrane Collaboration, [computer program]; 2014 software. First, the results of a single study will be described. Next, hazard ratios and 95% confidence intervals (CI) for dichotomous data are calculated separately. The relative risk (RR) and its 95% confidence interval (CI) will be used as the dichotomous outcome variable for a method of invigorating the spleen, supplementing qi, and tonifying kidney efficacy and safety; the mean difference (MD) and its 95% confidence interval will be used as the continuous variable to differentiate between groups the resulting values were compared to describe the therapeutic effect of method of the invigorating spleen, supplementing qi and tonifying kidney.

#### 2.7.2. Heterogeneity test.

To assess clinical heterogeneity between studies, similarities between studies will be assessed, including clinical heterogeneity of study subjects, interventions, controls, and outcome measures. In order to evaluate the statistical heterogeneity between studies, the statistical heterogeneity will be judged according to the results of *I*^2^ test; Among them, 0 < *I*^2^ < 50% means that the heterogeneity is small, and the fixed effect model can be used to combine statistics; 50%<*I*^2^ < 75% indicates high heterogeneity; *I*^2^ > 75% indicates great heterogeneity. Cochrane risk of bias tool (RoB 2.0) will be conducted: randomization process, deviations from the intended interventions, missing outcome data, outcome measurements, selection of the reported results, and overall bias according to the three criteria of “low risk,” “high risk” or “some concerns.”

#### 2.7.3. Sensitivity analysis.

In order to investigate the stability of the results, we will conduct a sensitivity analysis of the results. When significant statistical heterogeneity was found, we excluded low-quality trials, replicated meta-analysis papers, studies with insufficient sample size and/or data that were included in the analysis one by one, then reanalyzed and pooled the data, and compared the re-obtained difference between the effect and the original effect. In this way, we will be able to assess the impact of individual studies on the overall results, and whether the results are reliable.

#### 2.7.4. Publication bias.

Publication bias will be examined graphically and visually using a funnel plot and using Egger’s test, an approximate symmetry indicating the absence of publication bias.

#### 2.7.5. Subgroup analysis.

To obtain reliable data, subgroup analysis will be performed according to the duration of the herbal interventions for invigorating spleen and invigorating qi and tonifying kidney, drug dosage form, and MG typing.

#### 2.7..6. Quality of the evidence.

The quality of the evidence for the results of meta-analyses in relation to patient outcomes will be assessed using the Grading of Recommendations Assessment, Development and Evaluation (GRADE) criteria developed by the World Health Organization and international organizations.^[6]^ In terms of methodological quality, consistency of findings, directness and accuracy of evidence, and possibility of publication bias, we will judge the quality of evidence based on the level of evidence from RCT results. The GRADE system classifies the quality of evidence into 4 levels: high, moderate, low and very low.

### 2.8. Ethics and communication

As the data we used were secondary data, ethical approval or informed consent was not required for this systematic review. The scheme reviewed by the system will be published in peer reviewed journals and relevant national and international conferences.

## 3. Discussion

Modern medical treatment of MG often applies glucocorticoids, immunosuppressants, cholinesterase inhibitors, plasma replacement, and thymectomy to relieve clinical symptoms and delay disease progression. A large epidemiological study in China reported that the incidence and prevalence of MG were 0.155‐0.366 per 1 million people and 2.19‐11.07 per 1 million people, respectively.^[7]^ It is estimated to affect more than 700,000 people worldwide, and with the increasing aging of the population, the actual incidence is rising year by year.^[8]^ Studies have shown that herbs invigorating spleen and invigorating qi and tonifying kidney. Through the interaction between each herbal medicine, it can reduce the production of AchR-Ab, regulate the immune function of the body, downregulate the number of undifferentiated B lymphocytes and BAFF and IL-6 levels in peripheral blood, protect the skeletal muscle neuromuscular junction from damage in MG patients, and improve the symptoms of muscle weakness.^[9‐11]^ Experimental studies have shown that herbal medicines for invigorating spleen and invigorating qi and tonifying kidney can regulate the clinical symptoms of EAMG rats and reduced the levels of cellular inflammatory factors such as TNF-α, IL-4, and IL-17 in serum and inhibited the production of AchR-Ab.^[12,13]^ Therefore, this study will strictly follow the requirements of evidence-based medicine and use a large sample, high-quality RCT to evaluate the efficacy and safety of spleen-enhancing, qi-supplementing, and kidney-supplementing herbal medicine for MG, to provide clinicians and patients with evidence-based medical evidence and better to better serve the clinic.

## Author contributions

**Conceptualization:** Hongan Wang, Miao Liu.

**Data curation:** Hongan Wang, Miao Liu.

**Formal analysis:** Chaoqun Song, Ye Tian, Zhiyue Zhu.

**Funding acquisition:** Li Liu.

**Investigation:** Chunwei Wu, Zhiyue Zhu.

**Methodology:** Jing Lu, Zhiguo Lv.

**Supervision:** Dongmei Zhang, Jing Lu.

**Validation:** Tong Wu, Ye Tian.

**Writing – original draft:** Miao Liu.

**Writing – review & editing:** Li Liu, Xinzhi Chen.
